# There's an App for That: Development of an Application to Operationalize the Global Diet Quality Score

**DOI:** 10.1093/jn/nxab196

**Published:** 2021-10-23

**Authors:** Mourad Moursi, Sabri Bromage, Teresa T Fung, Sheila Isanaka, Mika Matsuzaki, Carolina Batis, Analí Castellanos-Gutiérrez, Erick Angulo, Nick Birk, Shilpa N Bhupathiraju, Yuna He, Yanping Li, Wafaie Fawzi, Armen Danielyan, Sachit Thapa, Liseteli Ndiyoi, Marieke Vossenaar, Alexandra Bellows, Joanne E Arsenault, Walter C Willett, Megan Deitchler

**Affiliations:** Intake – Center for Dietary Assessment, FHI Solutions, Washington DC, USA; Department of Nutrition, Global Health and Population, and Epidemiology, Harvard TH Chan School of Public Health, Boston, MA, USA; Department of Nutrition, Global Health and Population, and Epidemiology, Harvard TH Chan School of Public Health, Boston, MA, USA; Department of Nutrition, Global Health and Population, and Epidemiology, Harvard TH Chan School of Public Health, Boston, MA, USA; Department of International Health, Johns Hopkins Bloomberg School of Public Health, Baltimore, MD, USA; Health and Nutrition Research Center, National Institute of Public Health, Cuernavaca, Mexico; Health and Nutrition Research Center, National Institute of Public Health, Cuernavaca, Mexico; Health and Nutrition Research Center, National Institute of Public Health, Cuernavaca, Mexico; Department of Biostatistics, Harvard TH Chan School of Public Health, Boston, MA, USA; Department of Nutrition, Global Health and Population, and Epidemiology, Harvard TH Chan School of Public Health, Boston, MA, USA; Channing Division of Network Medicine, Department of Medicine, Brigham and Women's Hospital, Harvard Medical School, Boston, MA, USA; National Institute for Nutrition and Health, Chinese Center for Disease Control and Prevention, Beijing, China; Department of Nutrition, Global Health and Population, and Epidemiology, Harvard TH Chan School of Public Health, Boston, MA, USA; Department of Nutrition, Global Health and Population, and Epidemiology, Harvard TH Chan School of Public Health, Boston, MA, USA; Department of Epidemiology, Harvard TH Chan School of Public Health, Boston, MA, USA; Digital Development, FHI 360, Washington DC, USA; Digital Development, FHI 360, Washington DC, USA; Independent consultant, Lusaka, Zambia; Intake – Center for Dietary Assessment, FHI Solutions, Washington DC, USA; Department of International Health, Johns Hopkins Bloomberg School of Public Health, Baltimore, MD, USA; Intake – Center for Dietary Assessment, FHI Solutions, Washington DC, USA; Department of Nutrition, Global Health and Population, and Epidemiology, Harvard TH Chan School of Public Health, Boston, MA, USA; Channing Division of Network Medicine, Department of Medicine, Brigham and Women's Hospital, Harvard Medical School, Boston, MA, USA; Department of Epidemiology, Harvard TH Chan School of Public Health, Boston, MA, USA; Intake – Center for Dietary Assessment, FHI Solutions, Washington DC, USA

**Keywords:** GDQS, operationalization, data collection, application, sensitivity analysis

## Abstract

**Background:**

The global diet quality score (GDQS) is a simple, standardized metric appropriate for population-based measurement of diet quality globally.

**Objectives:**

We aimed to operationalize data collection by modifying the quantity of consumption cutoffs originally developed for the GDQS food groups and to statistically evaluate the performance of the operationalized GDQS relative to the original GDQS against nutrient adequacy and noncommunicable disease (NCD)-related outcomes.

**Methods:**

The GDQS application uses a 24-h open-recall to collect a full list of all foods consumed during the previous day or night, and automatically classifies them into corresponding GDQS food group. Respondents use a set of 10 cubes in a range of predetermined sizes to determine if the quantity consumed per GDQS food group was below, or equal to or above food group-specific cutoffs established in grams. Because there is only a total of 10 cubes but as many as 54 cutoffs for the GDQS food groups, the operationalized cutoffs differ slightly from the original GDQS cutoffs.

**Results:**

A secondary analysis using 5 cross-sectional datasets comparing the GDQS with the original and operationalized cutoffs showed that the operationalized GDQS remained strongly correlated with nutrient adequacy and was equally sensitive to anthropometric and other clinical measures of NCD risk. In a secondary analysis of a longitudinal cohort study of Mexican teachers, there were no differences between the 2 modalities with the beta coefficients per 1 SD change in the original and operationalized GDQS scores being nearly identical for weight gain (-0.37 and -0.36, respectively, *P* < 0.001 for linear trend for both models) and of the same clinical order of magnitude for waist circumference (-0.52 and -0.44, respectively, *P* < 0.001 for linear trend for both models).

**Conclusion:**

The operationalized GDQS cutoffs did not change the performance of the GDQS and therefore are recommended for use to collect GDQS data in the future.

## Introduction

Poor-quality diet is a leading cause of adverse health outcomes related to both undernutrition and overnutrition (1). Although many diet quality metrics have been developed and used, there is still not a widely used, relatively simple, and validated metric to measure diet quality [defined as both adequate in nutrients and protective against diet-related noncommunicable disease (NCD) risk outcomes] in population-based surveys in settings across the country (2). The Global Diet Quality Score (GDQS) was designed to fill this absence, thereby providing a simple, standardized metric appropriate for population-based measurement of diet quality globally. Following the success in developing and validating the GDQS (3), we sought to operationalize the metric and develop the necessary tools to collect GDQS data in a reliable and practical way in the context of large-scale surveys globally.

To facilitate the integration of GDQS in global monitoring frameworks and routine surveys in low- and middle-income countries (LMICs), the GDQS data collection tools should ideally: *1*) be easy to administer among low-literacy populations in LMICs; *2*) take no more than an average of 10 min per respondent to complete; and *3*) not require extensive or specialized training of enumerators. With these principles, we identified 2 central challenges to the operationalization of the GDQS. Since the GDQS was designed for global application, the first challenge was to determine how to ask about foods and beverages consumed in the previous day or night and classify them into the GDQS food groups in a quick and easy manner while requiring little adaptation to countries or regions. The classification of foods, beverages, and ingredients of mixed dishes represents a substantial burden on the respondent and/or the enumerator, which could affect not only the validity of collected data but also the comparability of the GDQS globally. The second challenge was determining how to collect information regarding quantity consumed in grams at the food group level to apply the GDQS’ group-specific cutoffs.

To address the first challenge, we developed a data collection application for collecting the GDQS data. For the second challenge and the inherent difficulty in collecting accurate information on the quantity of food consumed from respondents, we developed a simplified method for assessing the quantity of GDQS food group consumed by the respondent. The simplified method entails using a standard set of 10 3D cubes as visual aids to enable the respondent to easily classify the quantity of consumption per food group into the quantity of consumption categories (defined in grams) per GDQS food group.

To our knowledge, this is the first time that an application for global use has been developed for the collection of a diet quality metric that also captures the amounts of food groups consumed. The instrument used to collect the Minimal Diet Diversity Score for Women (MDD-W) excludes very small amounts of food (defined as <15 g, which represents for many foods ∼1 tablespoon) but otherwise does not attempt to estimate quantities consumed (4). The Diet Quality Questionnaire (DQ-Q) uses dichotomous yes/no questions to ask about the consumption of distinct food groups (5) and the parent instrument of the GDQS, called the Prime Diet Quality Score (PDQS), uses frequency data and does not directly estimate quantities consumed (6).

This paper describes the methods used to operationalize the GDQS and addresses the following questions: *1*) How does the application classify foods and beverages into the GDQS food groups? *2*) How was an average density value (g/cm^3^) for each of the GDQS food groups derived to translate the cutoffs into volumes to allow the use of cubes as visual aids? and *3*) Is the performance of the GDQS affected by the use of the operationalized cutoffs?

## Methods

### The application framework

The GDQS application was developed using an open-source Expo (7) framework, which is a set of tools and services built around React Native (8) used for the development, building, and deployment of applications. Although the GDQS application has only been tested to date on Android devices, the Expo framework allows compiling the source code of the application to work on iOS devices.

### Classification of foods and beverages into the GDQS food groups

**FIGURE 1:**
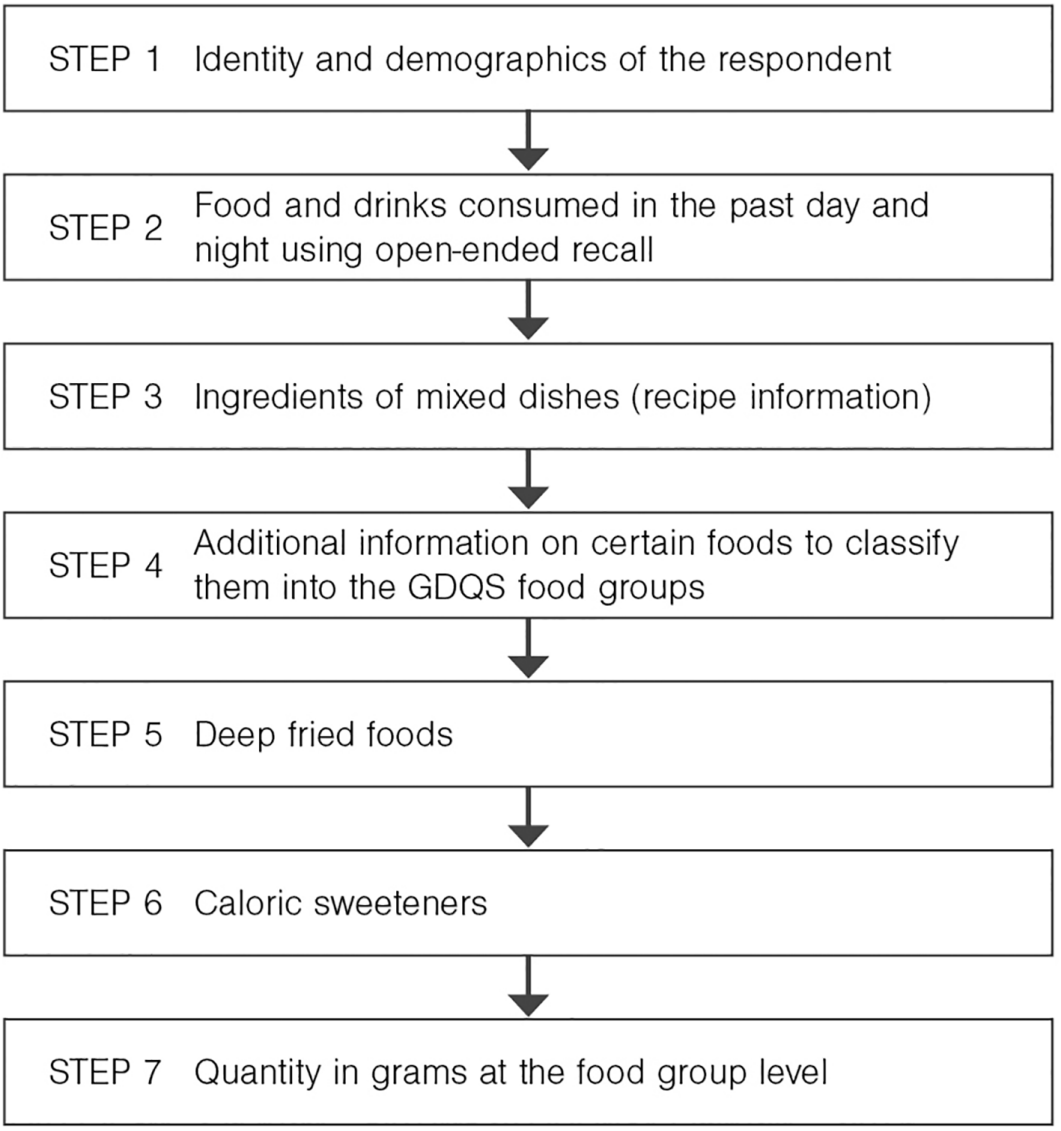
Data collection steps of the GDQS application. GDQS, Global Diet Quality Score.

The application proceeds in 7 steps to collect the GDQS data, with each step corresponding a different phase of the interview with the respondent (**Figure 1**). A video demonstration of the application is available at https://vimeo.com/515733474/8aa55d9350.

In the first step, the enumerator will obtain a list of all foods and beverages consumed during the preceding 24-h period by asking the respondent open-ended questions. To avoid underreporting and omission of foods, emphasis is put on the pattern of eating, going from one eating occasion to the next, including snacks in-between major eating occasions. This first part of the GDQS interview corresponds to the conventional first pass of a multipass 24-h recall (9).

Many foods reported are in the form of mixed dishes, which are typically defined as foods with a specific culinary name and prepared using ≥2 ingredients. In the GDQS application, when respondents report consuming a mixed dish, they are asked to list the foods that make up the mixed dish (i.e., the ingredients). If unable to recall the list of all ingredients of the mixed dish, they are asked to list only the main ingredients. There are some exceptions such as foods like bread and cakes, which are prepared with multiple ingredients but are treated as single foods rather than mixed dishes. The application is designed to capture all the information on reported foods or mixed dishes quickly and efficiently. Although the application allows for free text entry, it is designed to limit the need for free text by including a master database of foods, beverages, and mixed dishes compiled from food composition tables of West Africa (10), ASEAN (11), Kenya (12), Malawi (13), and India (14). In addition, the database includes foods and beverages in the FAO and Food and Nutrition Technical Assistance III Project MDD-W measurement guide (4). The master database currently has about 2700 entries of single foods and beverages with more entries to be added as we continue to prepare the GDSQ application for global release. The database is maintained in an Excel spreadsheet and can be edited to add missing foods and beverages in the upload of the database into the application before data collection in any setting begins. During the interview, the enumerator has the option to either select the food name from a drop-down menu, which can narrow choices down based on the first few letters typed in using approximate string matching (fuzzy search), or enter the food name in free text (if the food is missing from the master database).

Each food and beverage in the application master database is preclassified into its corresponding GDQS food group and the application uses that information to classify the foods, beverages, and ingredients of mixed dishes into the correct GDQS food group. If a food or ingredient is missing from the master database and the enumerator entered it using free text, the enumerator is responsible for manually classifying into 1 of the GDQS food groups displayed by the application before proceeding to the next step. As the master food database continues to grow, we anticipate that this will be an exceedingly rare occurrence.

Some foods, such as grains (whole or refined), dairy (high or low fat), and others may require more detailed information to be classified. The master database was therefore designed to include 2 additional fields with descriptors which are used dynamically by the application as probing questions. For example, the food “bread” in the list has an additional descriptor of either “white” or “brown.” The application uses that information to prompt the enumerator to ask the respondent if the bread reported as consumed was white or brown and the application then classifies the food accordingly either in the refined or whole grains GDQS food group.

The next step in the application asks about whether any of the foods reported as consumed were deep fried foods, with follow-up questions about whether these were purchased deep fried foods or were deep fried at home using pourable oil. There is an additional question asking whether the respondent poured oil on their food or used it to prepare foods. This step is followed by probing questions on the use of caloric sweeteners, which can be easily forgotten by respondents when reporting foods and beverages consumed. At the end of the steps described above, all foods and beverages will have been classified into their respective GDQS food groups.

### GDQS food group average density calculation

In its metric scoring approach, the GDQS assigns point values based on broad ranges of quantity of consumption (in g/d) at the food group level (3). Accounting for the quantity of consumption per GDQS food group poses a significant challenge for the respondent: to recall a reliable and valid estimate of the quantity is an extremely difficult mental exercise, especially at the food group level.

To address this challenge, data collection with the GDQS application entails the use of a set of 10 hollow 3D cubes in a range of predetermined sizes (**Supplemental Figure 1**). These cubes are used as visual aids for the respondent. The respondent is asked to visualize the quantity of all food items consumed within a GDQS food group and to indicate which cube size best represents the quantity consumed (i.e., volume). The application facilitates this process by instructing the enumerator to read back to the respondent the list of foods and beverages reported as consumed per GDQS food group. For example, if a person ate a banana in the morning and some watermelon in the evening, the enumerator would read those foods back to the respondent (both automatically classified under “other fruits” in the application) and ask the respondent to think about the combined volume of the banana and pieces of watermelon and choose the cube size which comes closest to the visualized volume.

This method of quantity estimation applies to all food groups except for “liquid oils” because, in most cases, it is unrealistic to expect respondents to recall the amount of liquid oils consumed. For this reason, the categorization of the amount of oil is inferred with the use of an algorithm that classifies the respondent above the highest range of oil consumption if *1*) they consumed deep fried foods prepared with liquid oil at home; *2*) they have had ≥2 mixed dishes; or *3*) they answer yes to the question of pouring liquid oil on their food or using it in food preparation. If the respondent has consumed only 1 mixed dish, the amount is classified in the middle range of oil consumption.

To operationalize this idea of using cubes as visual aids, we had to identify the size of the cube that is correct to associate with the given consumption cutoff (in grams) of a GDQS food group. For example, the original GDQS quantity cutoffs for red meat were <12, 12–48, and >48 g. In this case, the cutoffs of 12 and 48 g were used to help inform the sizes of the cubes. Converting a gram quantity consumption cutoff into a cube requires information on “average food group density.” To estimate the average food group densities, we used quantitative 24-h recall dietary datasets, prioritizing data from 10 LMICs given the focus on operationalizing the GDQS for use in these settings. In addition, we included 1 high-quality quantitative 24-h recall dietary dataset from a high-income country (HIC), to allow for the potential for the GDQS to also be used in HICs. The datasets used for this analysis are presented in **Table 1**. The choice of these datasets was driven by a combination of an established reputation for high quality, ease of accessibility, and representativeness of different regions of the world. The preference was to include nationally representative datasets, but we recognized that such data were not available in many LMICs. Therefore, we also included some datasets with a more limited statistical representativeness—often at the level of a few rural regions—that are known to be high quality. All the identified datasets included data on women of reproductive age (15–49 y of age) which were the reference age group for this analysis.

**TABLE 1:** 24-h dietary recall datasets used to compute average food group density^1^

Country/dataset name	Region	Year	Level
Mexico/ENSANUT	Latin America and the Caribbean	2016	National
Ethiopia	Sub-Saharan Africa	2019	National
Uganda HarvestPlus/A2Z	Sub-Saharan Africa	2006–2007	National
Zambia/HarvestPlus	Sub-Saharan Africa	2009	2 rural regions
Burkina Faso/IRD	Sub-Saharan Africa	2010	2 rural regions
India/HarvestPlus	South Asia region	2009–2011	Rural regions in the states of Punjab, Maharajtra and Gujarat
Bangladesh/BIHS	South Asia region	2016	National
Philippines	South-East Asia region	2013	National
Laos/PDR	South-East Asia region	2016–2017	National
China	East Asia	2010–2012	National
USA/NHANES	North America	2017–2018	National

1 BIHS, Bangladesh Integrated Household Survey; ENSANUT, Encuesta Nacional de Salud y Nutrición; IRD, Institut de Recherche pour le Développement; PDR, People Democratic Republic.

Though the GDQS metric was validated for nonpregnant and nonlactating women, we also used food consumption data for pregnant and lactating women to broaden the source of foods and increase sample size. We used only the first 24-h recall when >1 was available per individual.

In each dataset, foods were classified into 1 of the GDQS food groups, except for purchased deep fried foods. The deep fried foods, such as fried chicken, were classified in both their original group (in this example “poultry and game meat”) and in the purchased “deep fried foods” group. The “liquid oils” food group was excluded because the GDQS application does not directly ask about oil consumption. The percentage gram contribution of each individual food to the total of the food group was computed by dividing the gram sum of each individual food across all women by the total gram sum of the GDQS food group. Datasets contained primarily the amounts of raw foods and ingredients consumed from mixed dishes, which coincides with the way ingredients of mixed dishes are reported. For each dataset, we identified the top 5 foods in terms of percentage gram contribution to each GDQS food group and assigned ranks from 1 to 5. This resulted in a list of up to 55 foods having been identified per food group across all datasets (5 from each of 11 datasets). To keep the number of foods manageable for density compilation and computation, we narrowed down the list to a maximum of 10 foods for each food group by computing mean ranks for all identified foods and selecting the 10 foods with the lowest mean ranks across the 11 datasets (with 1 being the best rank). Those 10 foods with the lowest mean ranks were considered as the foods representing each GDQS food group globally (**Table 2****)**. For some GDQS food groups, such as deep orange tubers or eggs, there were <10 unique foods reported as consumed to be found across all datasets.

**TABLE 2:** Foods and beverages chosen to represent GDQS food groups and the corresponding mean food group density^1^

GDQS food group	Full list of foods and beverages chosen to represent the food group	Mean density (g/cm^3^)
Whole grains	Maize on cob, whole grain bread, whole maize grains, whole-grain tortilla, whole wheat roti/chapati, oats, whole flour injera, nshima, pearl millet roti/chapati	0.65
Refined grains and baked goods	White rice, white bread, pasta, biscuits, wheat roti/chapati, tortilla, sweet bread, refined flour injera, ugali, fritters	0.62
Deep orange tubers	Orange sweet potato	0.65
White roots and tubers	Potatoes, french fries, white sweet potatoes, plantain, cassava, yam, ginger, taro, yam bean	0.78
Legumes	Cowpeas, lentils, soybean, kidney beans, soymilk, peas, mung beans, black beans, pinto beans, chickpeas	0.80
Nuts and seeds	Peanuts/groundnuts, sesame seeds, peanut butter, almonds, sunflower seeds, jackfruit seeds, cowpea seeds, cacao seeds, Job's tears, cumin seeds	0.68
Fish and shellfish	Tilapia, tuna, catfish, carp, mullet, perch, mackerel, orangefin barb, sea fish, unspecified fish	0.57
Poultry and game meat	Chicken average cut, chicken leg, chicken breast, chicken wings, duck, fowl, buffalo, rat, chicken thigh, donkey	0.83
Red meat	Beef, pork, goat, mutton, liver, beef/goat stomach, beef entrails, pork ribs, lamb, pork organs	0.87
Processed meat	Smoked/dried beef, ham, pork sausage, turkey ham, burger meat, turkey sausage, dried pork, smoked/dried goat meat, chorizo, bratwurst	0.81
Eggs	Chicken eggs	0.6
High fat dairy: hard cheese	Hard cheese (unspecified), cheddar cheese	0.84
High fat dairy: other	Whole milk, sour milk, buffalo milk, whole-milk yogurt, curd, whole-milk drinkable yogurt, Oaxaca cheese, soft cheese	1.02
Low fat dairy	Low fat milk, low fat yogurt, skimmed buffalo milk, low fat curd, low fat cream cheese, low fat cheese spread	0.96
Deep orange fruits	Mango, papaya, cantaloupe, nanche, persimmon, tree tomato	0.73
Citrus fruits	Orange, lemon, tangerine, pomelo, grapefruit	0.71
Other fruits	Banana, watermelon, apple, avocado, jack fruit, grapes, green papaya, guava, pineapple	0.78
Dark green leafy vegetables	Spinach, sweet potato leaves, pumpkin leaves, cowpea leaves, amaranth leaves, kale, baobab leaves, moringa leaves, jute mallow leaves	0.70
Deep orange vegetables	Carrot, pumpkin	0.85
Cruciferous vegetables	Cabbage, broccoli, cauliflower, Chinese cabbage	0.67
Other vegetables	Tomato, onion, eggplant, water gourd, cucumber, okra, sweet peppers, fresh chili, zucchini, shallots	0.68
Liquid oils	NA	NA
Juice	Orange juice, apple juice, grape juice, fruit juice (unspecified), lemon juice, bissap, pineapple juice, cranberry juice, blackberry juice, roselle drink	1.05
Sugar-sweetened beverages	Soda/cola drink, sweet flavored water, energy drink, sports drink, industrial juice, flavored/chocolate milk, ovaltine, zoom komm, lassi	1.07
Sweets and ice cream	Sugar, cake, ice cream, sugarcane, honey, hard candy, jelly, cookies, sorghum cane, jaggery	0.70
Purchased deep fried foods	French fries, deep fried doughnuts, fritters, fried onion, chips, churro, fried chicken, deep fried spring roll, fried fish, fried cake	0.46

^1^GDQS, Global Diet Quality Score; NA, not applicable.

We compiled the density data using the FAO/INFOODS Density Database version 2.0 (15), the 2018 New Zealand food composition table (16), conversion factor databases associated with the datasets we used (Uganda, Burkina Faso, and India), and limited primary data collection with food items bought in stores. For each GDQS food group, the food group average density was computed using a simple mean of the densities of the foods representing the group.

Bearing in mind the purpose and kind of use the GDQS will be put to, the densities of foods within a given GDQS food group were deemed close enough to use simple means and in doing so, strike a compromise between ease of use and data precision. However, the high fat dairy group was the 1 exception where the densities of hard cheese and milk were so different that no reasonable compromise could be reached. Therefore, for operationalization purposes, we split the high fat dairy group into “hard cheese” and “other,” with other including mainly milk, yogurt, and soft cheese. This means that in practice, during data collection, the number of GDQS food groups temporarily increases to 26 and that of cutoffs to 54. The application asks about the consumed volume of hard cheese and other high fat dairy separately, but the 2 are combined again when reporting the GDQS results to align with the metric as originally validated with 25 food groups and 51 cutoffs. To combine them, the amount of hard cheese estimated using the cubes is multiplied by 6.1 (17) to convert it to milk equivalents.

### Food group quantity estimation and sensitivity analysis

Using the food group average densities, we converted the original 54 cutoffs of the 26 GDQS food groups (**Table 3**) into volumes of cubes. We produced plastic hollow 3D cubes using a 3D printer, with each cube representing an exact cutoff in grams. Given that using 54 cubes is not practical in field conditions, and because some differences in cube size were barely perceptible to the naked eye, we reduced the number of cubes to a total of 8 by grouping subsets of cubes that were similar in size and averaging their sizes. Two cubes, the smallest one and the largest one (both corresponding to no cutoffs), were added at each end of the set of 8 cubes to smooth out the visual representation of size increase and protect against a potential respondent desire to either report or not report the smallest or largest cube size, thus giving us a total of 10 cubes.

**TABLE 3:** Original and operationalized GDQS cutoffs and cube size, by food group^1^

GDQS food group	Cutoff range	Cube number	Cube side size (mm)	Original cutoff (g)	Operationalized cutoff (g)
No group (cube added at the beginning)	NA	1	18	NA	NA
Whole grains	Low/middle	2	22	4	8
Nuts and seeds	Low/middle	2	22	4	7
Processed meat	Low/middle	2	22	8	9
Refined grains and baked goods	Low/middle	2	22	7	7
Eggs	Low/middle	2	22	7	6
Deep orange vegetables	Low/middle	2	22	10	9
Legumes	Low/middle	2	22	10	9
Red meat	Low/middle	2	22	12	9
High fat dairy: hard cheese	Low/middle	2	22	35	9
Dark green leafy vegetables	Low/middle	3	27	10	13
Poultry and game meat	Low/middle	3	27	12	16
Sweets and ice cream	Low/middle	3	27	11	13
Cruciferous vegetables	Low/middle	3	27	11	13
Deep orange tubers	Low/middle	3	27	14	12
Purchased deep fried foods	Low/middle	3	27	10	9
Fish and shellfish	Low/middle	3	27	16	14
Whole grains	Middle/high	3	27	16	13
Nuts and seeds	Middle/high	3	27	16	13
Citrus fruits	Low/middle	4	32	18	24
White roots and tubers	Low/middle	4	32	25	27
Juice	Low/middle	4	32	35	36
Other fruits	Low/middle	4	32	26	27
Processed meat	Middle/high	4	32	31	30
High fat dairy: hard cheese	Middle/high	4	32	140	28
High fat dairy: other	Low/middle	4	32	35	35
Low fat dairy	Low/middle	4	32	35	33
Other vegetables	Low/middle	4	32	26	23
Deep orange fruits	Low/middle	4	32	28	25
Refined grains and baked goods	Middle/high	5	38	28	33
Deep orange vegetables	Middle/high	5	38	39	45
Eggs	Middle/high	5	38	28	32
Sugar sweetened beverage	Low/middle	5	38	52	57
Legumes	Middle/high	5	38	39	42
Red meat	Middle/high	5	38	48	46
Dark green leafy vegetables	Middle/high	5	38	39	37
Poultry and game meat	Middle/high	5	38	48	44
Sweets and ice cream	Middle/high	5	38	45	37
Cruciferous vegetables	Middle/high	5	38	44	36
Fish and shellfish	Middle/high	6	46	63	71
Purchased deep fried foods	Middle/high	6	46	40	45
Deep orange tubers	Middle/high	6	46	57	63
Citrus fruits	Middle/high	6	46	74	69
White roots and tubers	Middle/high	7	52	100	107
Juice	Middle/high	7	52	141	144
Other fruits	Middle/high	7	52	106	107
Low fat dairy	Middle/high	7	52	139	132
High fat dairy: hard cheese	High/very high	7	52	734	114
High fat dairy: other	Middle/high	7	52	140	143
Other vegetables	Middle/high	8	55	106	114
Deep orange fruits	Middle/high	8	55	114	123
Sugar sweetened beverage	Middle/high	8	55	207	180
High fat dairy: other	High/very high	9	89	734	734
No group (cube added at the end)	NA	10	100	NA	NA

1 When using the application, respondents use a set of 10 cubes in a range of predetermined sizes as visual aids to determine if the quantity consumed per GDQS food group was below, equal to, or above food group–specific cutoffs established in grams. Because there is only a total of 10 cubes (for practical reasons) but as many as 54 cutoffs for the GDQS food groups, the operationalized cutoffs differ slightly from the original GDQS cutoffs presented for the validation of the metric (3). For operationalization purposes, the high fat dairy group was divided into “hard cheese” and “other”, with “other” including mainly milk, yogurt, and soft cheese. This means that in practice, during data collection, the number of GDQS food groups temporarily increases to 26 and that of cutoffs to 54. The application asks about the consumed volume of hard cheese and other high fat dairy separately, but the 2 are combined again when reporting the GDQS results to align with the metric as originally validated with 25 food groups. GDQS, Global Diet Quality Score; NA, not applicable.

2 For each GDQS food group, the values below the smallest cutoff in grams were defined as “low”, values falling in between the smallest and largest cutoffs were defined as “middle”, and values above the largest cutoff were defined as “high”. The high fat dairy group had an additional cutoff compared with the rest of the food groups and value above the largest cutoff in grams were defined as very high. See Table 3 in Bromage et al (3).

Because there is only a total of 10 cubes but as many as 54 cutoffs for the 25 GDQS food groups, the operationalized cutoffs differ slightly from the original cutoffs presented for the validation of the metric. The operationalized and the original cutoffs are presented in Table 3.

It is important to note that sometimes the size of given cube could correspond to the exact cutoff of a GDQS food group. In those borderline cases where the respondent reports consuming an amount (i.e., select a cube size) that corresponds to the exact cutoff, the application is programmed to prompt her with a follow-up question asking if the amount visualized for the food group is “as big or bigger” or “smaller” than the designated cube, as a way of confirming the information.

We conducted a sensitivity analysis comparing the performance of GDQS using the operationalized and original cutoffs on the following cross-sectional datasets used in the original GDQS validation research (3): Ethiopia, China, Mexico, India, and the Millennium Villages Project (MVP). One longitudinal cohort study dataset used in the original GDQS validation research (3), the Mexican Teachers Cohort (MTC), was also used. We compared covariate-adjusted associations between the metrics and energy-adjusted aggregate measures of protein, fiber, calcium, iron, zinc, vitamin A, folate, and vitamin B12 adequacy. In all cross-sectional datasets except China, nutrient adequacy was used as a continuous variable and estimated marginal means are presented. In China, nutrient (in)adequacy was defined as the mean probability of adequacy for the 9 nutrients presented below 50%, and the OR is presented. For cross-sectional data, regression models present changes in nutrient adequacy and NCD-risk outcomes per 1-SD increase in the GDQS score and associated *P* values for linear trends across quintiles of the GDQS metrics. Analyses with cohort data from MTC present the results of the association of 2-y change in metrics and 2-y change in weight and waist circumference using generalized linear models. We conducted Wald tests between the original GDQS and the operationalized GDQS to detect any statistically significant differences in performance between the 2 metrics.

## Results

Select results that were representative of the performance difference between the 2 metrics in predicting energy-adjusted nutrient adequacy and NCD outcomes are presented in **Table 4** for the cross-sectional data and **Table 5** for the cohort data. The full results are available in **Supplemental Table 1**. Overall, there were no differences in performance between the 2 metrics. In the cross-sectional datasets, the operationalized GDQS remained strongly correlated with nutrient adequacy. The largest observed difference between the original and operationalized GDQS in the coefficient of estimated marginal means of nutrient adequacy per 1 SD of GDQS score was 0.07 in the Mexico cross-sectional data but it was not statistically significant (*P* = 0.12). Compared with the original GDQS, the operationalized GDQS was equally sensitive to clinical measures of NCD risk and anthropometry with no change in the OR coefficients per 1 SD of the GDQS score for metabolic syndrome (China), BMI (kg/m^2^) ≥25 (Ethiopia and MVP), total cholesterol (Mexico), or HDL cholesterol ≤50 mg/dL (India). In an analysis of the longitudinal MTC, the 2 metrics performed equally well with the beta coefficients per 1-SD change in the original and operationalized scores being nearly identical for weight gain (-0.37 and -0.36, respectively) and of the same clinical order of magnitude for waist circumference (-0.52 and -0.44, respectively) over a period of 2 y.

**TABLE 4:** Select operationalized compared with original GDQS associations with outcome categories of nutrient adequacy and measures of NCD risk using cross sectional data^1^

Dataset	Outcome	Statistic	*n*	GDQS	*β* coefficient per 1 SD (95% CI)	*P* value, trend^2^	*P* value, difference^3^
Nutrient adequacy^4^							
Ethiopia	Nutrient adequacy	Mean	1604	Operationalized/original	0.28 (0.21, 0.35)/0.32 (0.25, 0.39)	<0.001/<0.001	0.08
Mexico	Nutrient adequacy	Mean	2467	Operationalized/original	2.17 (1.81, 2.52)/2.24 (1.89, 2.59)	<0.001/<0.001	0.12
China	Nutrient inadequacy	OR	14,938	Operationalized/original	0.38 (0.36, 0.40)/0.41 (0.39, 0.44)	<0.001/<0.001	NA^5^
India	Nutrient adequacy	Mean	3041	Operationalized/original	0.25 (0.21, 0.28)/0.28 (0.25, 0.31)	<0.001/<0.001	0.29
MVP	Nutrient adequacy	Mean	1624	Operationalized/original	0.46 (0.39, 0.53)/0.51 (0.45, 0.58)/	<0.001/<0.001	0.17
NCD risk							
Ethiopia	BMI ≥25	OR	1596	Operationalized/original	1.20 (1.02, 1.41)/1.19 (1.02, 1.40)	0.008/0.054	0.28
Mexico	Total cholesterol (mg/dL)	Mean	1513	Operationalized/original	−2.81 (−4.55, −1.07)/−2.91 (−4.65, −1.16)	0.006/0.003	0.38
China	Metabolic syndrome	OR	11,148	Operationalized/original	0.87 (0.82, 0.92)/0.88 (0.83, 0.93)	<0.001/<0.001	NA^5^
India	HDL cholesterol ≤50 mg/dL	OR	3041	Operationalized/original	1.16 (1.07, 1.26)/1.12 (1.04, 1.22)	0.001/0.002	0.62
MVP	BMI ≥25	OR	451	Operationalized/original	0.99 (0.77, 1.27)/1.06 (0.82, 1.36)	0.72/0.74	0.80

1 Data presented for Ethiopia, India, and MVP are FFQ data; Mexico and China are 24-h recall data. GDQS, Global Diet Quality Score; MVP, Millennium Villages Project; NA, not applicable; NCD, noncommunicable disease.

2 *P* value, trend is presented for quintile analysis.

3 Wald test results for difference between original and operationalized GDQS.

4 Nutrient adequacy is an Estimated Average Requirement–based, energy-adjusted aggregate measure of protein, fiber, calcium, iron, zinc, vitamin A, folate, and vitamin B12. In all datasets except China, nutrient adequacy was used as a continuous variable and estimated marginal means are presented. In China, nutrient inadequacy was defined as the mean probability of adequacy for the 9 nutrients presented below 50% and OR is presented.

5 In the China dataset, the scores for the operationalized and original GDQS were so colinear that it was not possible to derive a meaningful test for difference between the 2 scores.

**TABLE 5:** Beta coefficients for the association of operationalized and original GDQS with weight change and waist circumference change in the Mexican Teachers Cohort^1^

	Largest decrease	Small decrease	Little change	Small increase	Largest increase	Per 1 SD	Wald test^2^	*P*-trend^3^
GDQS score change	−5	−5 to −2	−2 to +2	>+2 to +5	>+5			
Weight change, kg								
GDQS	0.50 (0.19, 0.81)	0.33 (0.09, 0.57)	Reference	−0.43 (−0.67, −0.20)	−0.81 (−1.11, −0.51)	−0.37 (−0.47, −0.27)	0.19	<0.001
Operationalized GDQS	0.31 (0.01, 0.60)	0.36 (0.12, 0.60)	Reference	−0.33 (−0.57, −0.09)	−0.72 (−1.01, −0.43)	−0.36 (−0.46, −0.26)		<0.001
Waist circumference change, cm								
GDQS	0.54 (0.04, 1.12)	0.24 (−0.19, 0.69)	Reference	−0.49 (−0.93, −0.05)	−0.99 (−1.53, −0.45)	−0.52 (−0.71, −0.33)	0.91	<0.001
Operationalized GDQS	0.64 (0.06, 1.22)	0.32 (−0.13, 0.78)	Reference	−0.27 (−0.73, 0.17)	−1.04 (−1.59, −0.49)	−0.44 (−0.63, −0.25)		<0.001

1 Values are beta coefficients (95% CI) for weight and waist circumference change over 2 y. GDQS: 8967 women were included in weight change analysis; 7588 women were included in waist circumference analysis. Beta coefficients were adjusted for baseline age (continuous); change in energy (continuous); state (Jalisco, Veracruz); baseline GDQS score (continuous); 2006 and 2008 physical activity (low, medium, high); baseline marital status (single, living together, married, separated, widow); baseline education (none, ≤high school, undergraduate degree, ≥graduate degree); baseline household assets (low, middle, high); baseline health insurance (public, private, other); baseline BMI (< 25, 25–29.9, > 30); and changes in smoking status (baseline past smoker, stayed nonsmoker, stayed smokers, quitters, starters) and alcohol consumption (baseline nondrinkers, stayed nondrinkers, stayed drinkers, quitters, starters). GDQS, Global Diet Quality Score.

2 Wald test results between GDQS and operationalized GDQS.

3 Medians were fitted in a multivariate model to estimate *P*-trend.

## Discussion

The GDQS application includes a global database of 2700 foods from all regions of the world where foods and beverages are preclassified into corresponding GDQS food groups. This standardization helps to address some of the key challenges that have been reported with other methods commonly used for collecting data for food-based indicators in LMICs. These include the difficulty of adapting a list-based food group questionnaire to appropriately reflect the foods and beverages commonly consumed in the survey area, the difficulty of respondents to correctly report and classify foods and beverages consumed into the food groups included in a list-based food group questionnaire, and the difficulty of enumerators or other survey staff to appropriately classify foods and beverages reported as consumed into the correct corresponding food group when an open-recall method (as opposed to a list-based food group questionnaire) is used for data collection.

The use of this newly developed application does not require enumerators to have specific training or expertise in nutrition or food preparation in a given context to collect high-quality data to tabulate the GDQS and its submetrics, and that in-depth country-specific adaptation of the data collection instrument should not be required. Using enumerators to conduct the interviews with the help of the application facilitates data collection in contexts where targeted respondents may have a low level of literacy. However, in contexts with widespread high literacy and where personal ownership of tablet computers and phones is quite common, a self-administered version of the application may also be possible.

Use of the application also has the benefit of promoting standardized data collection and standardized classification of foods and beverages into the correct GDQS food group across all countries where data are collected. In addition, the open-recall method used for data collection allows for the individual foods and beverages reported as consumed by respondents to be retained as a rich source of data available for further analyses and use—a possibility pre-empted by the use of a limited list-based food group questionnaire. The collection of the quantity of consumption data needed to tabulate the GDQS will also be facilitated by use of the application. Because the application automatically classifies each food and beverage reported as consumed into the correct GDQS food group, enumerators will be equipped to read back to the respondent the list of foods and beverages consumed per GDQS food group when collecting information about the total quantity of foods and beverages consumed (in grams) by GDQS food group.

The main limitation of this work is that none of the tools and solutions developed have yet been tested in a field setting. Although 3D models such as wooden spheres or cubes have been used to estimate amounts of single foods and mixed dishes consumed in 24-h recall surveys, to our knowledge, they have not been used to estimate the aggregate amounts at the food group level. Whether respondents can successfully visualize amounts at the group level requires further testing and validation. This may be especially challenging when several different foods are consumed within a food group and when shared plate eating is widespread. In preparation for the global release of GDQS, validation studies to confirm the potential of these different options and tools to facilitate the collection of high-quality GDQS data and to estimate their ease of use and cost relative to other methods are needed.

The application will be made freely available for global public use and integration into existing electronic data collection systems, to ensure that the collection of the data required for the GDQS can be easily incorporated into population-based surveys globally. The application user interface is already available in English and French with more languages to come. There is currently no precise estimate of how much time per respondent is required to complete the interview, but preliminary internal testing indicated that the application could require approximately 10 min per respondent to complete, with the time required for data collection per respondent largely dependent on the pace of the interview and the complexity of the diet on the reference day of recall.

## Supplementary Material

nxab196_Supplemental_Figure_1

nxab196_Supplemental_Table_1

## Notes

Funding for the research was provided by a Bill & Melinda Gates Foundation grant to FHI Solutions to support Intake – Center for Dietary Assessment.

Author disclosures: TTF is an Associate Editor on the Journal of Nutrition and played no role in the Journal's evaluation of the manuscript. All other authors report no conflict of interest.

Published in a supplement to the Journal of Nutrition. Publication costs for this supplement were funded by the Bill & Melinda Gates Foundation in conjunction with FHI Solutions, recipient of a Bill & Melinda Gates Foundation grant to support Intake – Center for Dietary Assessment. The opinions expressed in this publication are those of the authors and are not attributable to the sponsors or the publisher, Editor, or Editorial Board of the *Journal of Nutrition*. The Supplement Coordinators for the supplement publication were Megan Deitchler, Intake – Center for Dietary Assessment at FHI Solutions, Washington, DC; and Sabri Bromage, Harvard T.H. Chan School of Public Health, Boston, MA.

Supplemental Figure 1 and Supplemental Table 1 are available from the “Supplementary data” link in the online posting of the article and from the same link in the online table of contents at https://academic.oup.com/jn.

The GDQS research initiative was launched by Intake – Center for Dietary Assessment. The research was led by Harvard T.H. Chan School of Public Health, Department of Nutrition and carried out in collaboration with researchers at the National Public Health Institute (INSP), Mexico. Funding for the research was provided by FHI Solutions, recipient of a Bill & Melinda Gates Foundation grant to support Intake – Center for Dietary Assessment.

Abbreviations used: DQ-Q, Diet Quality Questionnaire; GDQS, Global Diet Quality Score; HIC, high income country; LMIC, low- and middle-income country; MDD-W, Minimal Dietary Diversity score for Women; MVP, Millennium Villages Project; NCD, noncommunicable disease; PDQS, Prime Diet Quality Score.
