# Supplementary material for: There's an App for That: Development of an Application to Operationalize the Global Diet Quality Score
Source: J Nutr. 2021 Oct 23;151(Suppl 2):176S–184S. doi: 10.1093/jn/nxab196 (PMC8542098; doi:10.1093/jn/nxab196)

There's an app for that: development of an application to operationalize the Global Diet Quality Score. Mourad Moursi. Online Supplementary Material.

Supplemental Figure 1. Illustration of the 10 cubes

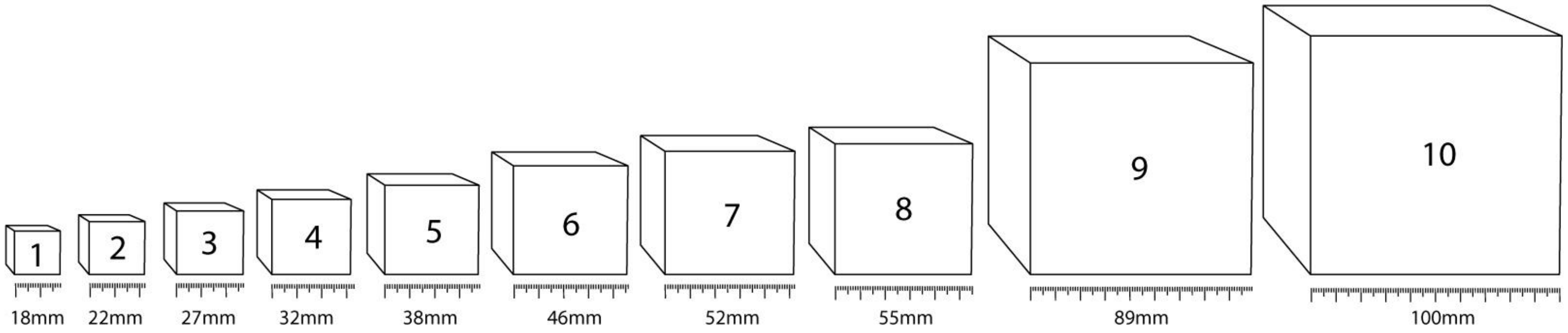

Supplement: nxab196_Supplemental_Figure_1 [file nxab196_supplemental_figure_1.pdf]
