# Supplementary material for: There's an App for That: Development of an Application to Operationalize the Global Diet Quality Score
Source: J Nutr. 2021 Oct 23;151(Suppl 2):176S–184S. doi: 10.1093/jn/nxab196 (PMC8542098; doi:10.1093/jn/nxab196)
Supplement: nxab196_Supplemental_Table_1 [file nxab196_supplemental_table_1.docx]

There's an app for that: development of a mobile application to operationalize the Global Diet Quality Score. Mourad Moursi. Online supplementary material.

Supplemental table 1. Operationalized vs original GDQS’ associations with nutrient adequacy and measures of NCD risk using cross sectional data

| **Dataset** | **Outcome** | **Statistic** | **n** | **Metric** | **Per 1 SD** | **95%CI** | | ***P*,**  **trend** | ***P*,**  **difference** |
| --- | --- | --- | --- | --- | --- | --- | --- | --- | --- |
| AnemEE | Body mass index (kg/m^2^) | Estimated marginal mean | 1596 | Operationalized GDQS | 0.35 | 0.18 | 0.53 | <0.001 | 0.294 |
| AnemEE | Body mass index (kg/m^2^) | Estimated marginal mean | 1596 | GDQS | 0.35 | 0.17 | 0.53 | <0.001 |  |
| AnemEE | Mid-upper arm circumference (cm) | Estimated marginal mean | 1596 | Operationalized GDQS | 0.29 | 0.13 | 0.46 | <0.001 | 0.301 |
| AnemEE | Mid-upper arm circumference (cm) | Estimated marginal mean | 1596 | GDQS | 0.31 | 0.15 | 0.48 | 0.003 |  |
| AnemEE | BMI ≥25 kg/m^2^ | Odds ratio | 1596 | Operationalized GDQS | 1.20 | 1.02 | 1.41 | 0.008 | 0.277 |
| AnemEE | BMI ≥25 kg/m^2^ | Odds ratio | 1596 | GDQS | 1.19 | 1.02 | 1.40 | 0.054 |  |
| AnemEE | Diastolic blood pressure (mmHg) | Estimated marginal mean | 1604 | Operationalized GDQS | 0.36 | -0.14 | 0.87 | 0.134 | 0.308 |
| AnemEE | Diastolic blood pressure (mmHg) | Estimated marginal mean | 1604 | GDQS | 0.53 | 0.03 | 1.03 | 0.044 |  |
| AnemEE | Hypertension (130/85 criteria) | Odds ratio | 1604 | Operationalized GDQS | 1.07 | 0.96 | 1.19 | 0.253 | 0.644 |
| AnemEE | Hypertension (130/85 criteria) | Odds ratio | 1604 | GDQS | 1.08 | 0.97 | 1.21 | 0.317 |  |
| AnemEE | Systolic blood pressure (mmHg) | Estimated marginal mean | 1604 | Operationalized GDQS | 1.22 | 0.45 | 1.99 | 0.002 | 0.729 |
| AnemEE | Systolic blood pressure (mmHg) | Estimated marginal mean | 1604 | GDQS | 1.46 | 0.69 | 2.23 | 0.001 |  |
| AnemEE | BMI <18.5 kg/m^2^ | Odds ratio | 1596 | Operationalized GDQS | 0.91 | 0.81 | 1.02 | 0.013 | 0.820 |
| AnemEE | BMI <18.5 kg/m^2^ | Odds ratio | 1596 | GDQS | 0.88 | 0.78 | 0.99 | 0.043 |  |
| AnemEE | Ferritin (ng/mL) | Estimated marginal mean | 782 | Operationalized GDQS | -0.34 | -3.94 | 3.26 | 0.634 | 0.811 |
| AnemEE | Ferritin (ng/mL) | Estimated marginal mean | 782 | GDQS | 0.30 | -3.33 | 3.93 | 0.648 |  |
| AnemEE | Ferritin <15 µg/L | Odds ratio | 782 | Operationalized GDQS | 0.98 | 0.82 | 1.17 | 0.690 | 0.264 |
| AnemEE | Ferritin <15 µg/L | Odds ratio | 782 | GDQS | 0.94 | 0.79 | 1.13 | 0.829 |  |
| AnemEE | Hemoglobin (g/dL) | Estimated marginal mean | 1485 | Operationalized GDQS | 0.07 | 0.00 | 0.14 | 0.062 | 0.495 |
| AnemEE | Hemoglobin (g/dL) | Estimated marginal mean | 1485 | GDQS | 0.08 | 0.01 | 0.15 | 0.034 |  |
| AnemEE | Hemoglobin <12 g/dL | Odds ratio | 1485 | Operationalized GDQS | 0.85 | 0.74 | 0.98 | 0.037 | 0.514 |
| AnemEE | Hemoglobin <12 g/dL | Odds ratio | 1485 | GDQS | 0.84 | 0.73 | 0.97 | 0.027 |  |
| AnemEE | Mid-upper arm circumference <24.5 cm | Odds ratio | 1596 | Operationalized GDQS | 0.86 | 0.77 | 0.95 | 0.001 | 0.512 |
| AnemEE | Mid-upper arm circumference <24.5 cm | Odds ratio | 1596 | GDQS | 0.84 | 0.76 | 0.93 | 0.006 |  |
| AnemEE | Overall nutrient adequacy (EAR-based) (energy-adjusted) | Estimated marginal mean | 1604 | Operationalized GDQS | 0.28 | 0.21 | 0.35 | <0.001 | 0.077 |
| AnemEE | Overall nutrient adequacy (EAR-based) (energy-adjusted) | Estimated marginal mean | 1604 | GDQS | 0.32 | 0.25 | 0.39 | <0.001 |  |
| AnemEE | Overall nutrient inadequate (EAR-based) (energy-adjusted) | Odds ratio | 1604 | Operationalized GDQS | 0.69 | 0.61 | 0.77 | <0.001 | 0.098 |
| AnemEE | Overall nutrient inadequate (EAR-based) (energy-adjusted) | Odds ratio | 1604 | GDQS | 0.65 | 0.57 | 0.73 | <0.001 |  |
| AnemEE | Serum B12 (pmol/L) | Estimated marginal mean | 1604 | Operationalized GDQS | 6.49 | -7.75 | 20.73 | 0.297 | 0.351 |
| AnemEE | Serum B12 (pmol/L) | Estimated marginal mean | 1604 | GDQS | 1.79 | -12.58 | 16.15 | 0.478 |  |
| AnemEE | Serum B12 <270 pmol/L | Odds ratio | 1604 | Operationalized GDQS | 1.01 | 0.87 | 1.17 | 0.703 | 0.266 |
| AnemEE | Serum B12 <270 pmol/L | Odds ratio | 1604 | GDQS | 1.08 | 0.93 | 1.25 | 0.408 |  |
| AnemEE | Serum folate (ng/mL) | Estimated marginal mean | 1604 | Operationalized GDQS | 0.42 | 0.15 | 0.69 | 0.002 | 0.429 |
| AnemEE | Serum folate (ng/mL) | Estimated marginal mean | 1604 | GDQS | 0.48 | 0.21 | 0.74 | 0.001 |  |
| AnemEE | Serum folate <4 ng/ml | Odds ratio | 1604 | Operationalized GDQS | 0.77 | 0.66 | 0.90 | 0.002 | 0.619 |
| AnemEE | Serum folate <4 ng/ml | Odds ratio | 1604 | GDQS | 0.76 | 0.65 | 0.89 | <0.001 |  |
| CNNHS | Metabolic syndrome | Odds ratio | 11148 | GDQS | 0.88 | 0.83 | 0.93 | <0.001 | <0.001 |
| CNNHS | Metabolic syndrome | Odds ratio | 11148 | Operationalized GDQS | 0.87 | 0.82 | 0.92 | <0.001 |  |
| CNNHS | Overall nutrient inadequate (probability-based) (energy-adjusted) | Odds ratio | 14938 | GDQS | 0.41 | 0.39 | 0.44 | <0.001 | <0.001 |
| CNNHS | Overall nutrient inadequate (probability-based) (energy-adjusted) | Odds ratio | 14938 | Operationalized GDQS | 0.38 | 0.36 | 0.40 | <0.001 |  |
| ENSANUT 24-hour recall | Overall nutrient adequacy (probability-based) (energy-adjusted) | Estimated marginal mean | 2467 | GDQS | 2.24 | 1.89 | 2.59 | <0.001 | 0.119 |
| ENSANUT 24-hour recall | Overall nutrient adequacy (probability-based) (energy-adjusted) | Estimated marginal mean | 2467 | Operationalized GDQS | 2.17 | 1.81 | 2.52 | <0.001 |  |
| ENSANUT 24-hour recall | Overall nutrient inadequate (probability-based) (energy-adjusted) | Odds ratio | 2467 | GDQS | 0.58 | 0.65 | 0.75 | <0.001 | 0.123 |
| ENSANUT 24-hour recall | Overall nutrient inadequate (probability-based) (energy-adjusted) | Odds ratio | 2467 | Operationalized GDQS | 0.59 | 0.53 | 0.66 | <0.001 |  |
| ENSANUT FFQ | Body mass index (kg/m^2^) | Estimated marginal mean | 4865 | GDQS | -0.25 | -0.41 | -0.08 | 0.018 | 0.277 |
| ENSANUT FFQ | Body mass index (kg/m^2^) | Estimated marginal mean | 4865 | Operationalized GDQS | -0.24 | -0.40 | -0.08 | 0.008 |  |
| ENSANUT FFQ | Waist circumference (cm) | Estimated marginal mean | 4213 | GDQS | -0.81 | -1.31 | -0.31 | 0.009 | 0.244 |
| ENSANUT FFQ | Waist circumference (cm) | Estimated marginal mean | 4213 | Operationalized GDQS | -0.83 | -1.33 | -0.33 | 0.003 |  |
| ENSANUT FFQ | BMI ≥25 kg/m^2^ | Odds ratio | 4865 | GDQS | 0.90 | 0.84 | 0.96 | 0.007 | 0.525 |
| ENSANUT FFQ | BMI ≥25 kg/m^2^ | Odds ratio | 4865 | Operationalized GDQS | 0.92 | 0.86 | 0.98 | 0.015 |  |
| ENSANUT FFQ | Total cholesterol (mg/dL) | Estimated marginal mean | 1513 | GDQS | -2.91 | -4.65 | -1.16 | 0.003 | 0.377 |
| ENSANUT FFQ | Total cholesterol (mg/dL) | Estimated marginal mean | 1513 | Operationalized GDQS | -2.81 | -4.55 | -1.07 | 0.006 |  |
| ENSANUT FFQ | Overall nutrient adequacy (EAR-based) (energy-adjusted) | Estimated marginal mean | 4868 | GDQS | 0.62 | 0.57 | 0.66 | <0.001 | 0.116 |
| ENSANUT FFQ | Overall nutrient adequacy (EAR-based) (energy-adjusted) | Estimated marginal mean | 4868 | Operationalized GDQS | 0.62 | 0.58 | 0.66 | <0.001 |  |
| ENSANUT FFQ | Overall nutrient inadequate (EAR-based) (energy-adjusted) | Odds ratio | 4868 | GDQS | 0.51 | 0.47 | 0.54 | <0.001 | 0.225 |
| ENSANUT FFQ | Overall nutrient inadequate (EAR-based) (energy-adjusted) | Odds ratio | 4868 | Operationalized GDQS | 0.51 | 0.48 | 0.54 | <0.001 |  |
| IMS/APCAPS | Body mass index (kg/m^2^) | Estimated marginal mean | 2961 | GDQS | 0.66 | 0.52 | 0.80 | <0.001 | 0.140 |
| IMS/APCAPS | Body mass index (kg/m^2^) | Estimated marginal mean | 2961 | Operationalized GDQS | 0.76 | 0.62 | 0.90 | <0.001 |  |
| IMS/APCAPS | Mid-upper arm circumference (cm) | Estimated marginal mean | 3030 | GDQS | 0.50 | 0.38 | 0.61 | <0.001 | 0.170 |
| IMS/APCAPS | Mid-upper arm circumference (cm) | Estimated marginal mean | 3030 | Operationalized GDQS | 0.55 | 0.43 | 0.66 | <0.001 |  |
| IMS/APCAPS | Waist circumference (cm) | Estimated marginal mean | 3035 | GDQS | 1.81 | 1.46 | 2.16 | <0.001 | 0.220 |
| IMS/APCAPS | Waist circumference (cm) | Estimated marginal mean | 3035 | Operationalized GDQS | 1.95 | 1.60 | 2.30 | <0.001 |  |
| IMS/APCAPS | BMI ≥25 kg/m^2^ | Odds ratio | 2961 | GDQS | 1.45 | 1.31 | 1.59 | <0.001 | 0.44 |
| IMS/APCAPS | BMI ≥25 kg/m^2^ | Odds ratio | 2961 | Operationalized GDQS | 1.52 | 1.38 | 1.68 | <0.001 |  |
| IMS/APCAPS | BMI ≥30 kg/m^2^ | Odds ratio | 2961 | GDQS | 1.51 | 1.27 | 1.80 | <0.001 | 0.65 |
| IMS/APCAPS | BMI ≥30 kg/m^2^ | Odds ratio | 2961 | Operationalized GDQS | 1.51 | 1.28 | 1.79 | <0.001 |  |
| IMS/APCAPS | Diastolic blood pressure (mmHg) | Estimated marginal mean | 3039 | GDQS | 0.09 | -0.28 | 0.45 | 0.613 | 0.190 |
| IMS/APCAPS | Diastolic blood pressure (mmHg) | Estimated marginal mean | 3039 | Operationalized GDQS | 0.31 | -0.05 | 0.68 | 0.180 |  |
| IMS/APCAPS | Diastolic blood pressure >85 mmHg | Odds ratio | 3039 | GDQS | 0.98 | 0.89 | 1.08 | 0.974 | 0.140 |
| IMS/APCAPS | Diastolic blood pressure >85 mmHg | Odds ratio | 3039 | Operationalized GDQS | 1.04 | 0.94 | 1.15 | 0.345 |  |
| IMS/APCAPS | Glucose (mg/dL) | Estimated marginal mean | 2958 | GDQS | 0.46 | -0.26 | 1.17 | 0.326 | 0.350 |
| IMS/APCAPS | Glucose (mg/dL) | Estimated marginal mean | 2958 | Operationalized GDQS | 0.40 | -0.31 | 1.12 | 0.351 |  |
| IMS/APCAPS | Glucose ≥100 mg/dL | Odds ratio | 2958 | GDQS | 1.00 | 0.90 | 1.11 | 0.639 | 0.630 |
| IMS/APCAPS | Glucose ≥100 mg/dL | Odds ratio | 2958 | Operationalized GDQS | 0.99 | 0.89 | 1.09 | 0.943 |  |
| IMS/APCAPS | HDL cholesterol (mg/dL) | Estimated marginal mean | 3041 | GDQS | -0.82 | -1.23 | -0.41 | <0.001 | 0.570 |
| IMS/APCAPS | HDL cholesterol (mg/dL) | Estimated marginal mean | 3041 | Operationalized GDQS | -0.93 | -1.34 | -0.52 | <0.001 |  |
| IMS/APCAPS | HDL cholesterol <50 mg/dL | Odds ratio | 3041 | GDQS | 1.12 | 1.04 | 1.22 | 0.002 | 0.620 |
| IMS/APCAPS | HDL cholesterol <50 mg/dL | Odds ratio | 3041 | Operationalized GDQS | 1.16 | 1.07 | 1.26 | 0.001 |  |
| IMS/APCAPS | Systolic blood pressure (mmHg) | Estimated marginal mean | 3039 | GDQS | 0.01 | -0.43 | 0.46 | 0.831 | 0.200 |
| IMS/APCAPS | Systolic blood pressure (mmHg) | Estimated marginal mean | 3039 | Operationalized GDQS | 0.26 | -0.18 | 0.71 | 0.302 |  |
| IMS/APCAPS | Systolic blood pressure >130 mmHg | Odds ratio | 3039 | GDQS | 1.03 | 0.91 | 1.15 | 0.738 | 0.170 |
| IMS/APCAPS | Systolic blood pressure >130 mmHg | Odds ratio | 3039 | Operationalized GDQS | 1.08 | 0.96 | 1.21 | 0.137 |  |
| IMS/APCAPS | Total cholesterol (mg/dL) | Estimated marginal mean | 2956 | GDQS | 3.36 | 1.82 | 4.90 | <0.001 | 0.220 |
| IMS/APCAPS | Total cholesterol (mg/dL) | Estimated marginal mean | 2956 | Operationalized GDQS | 3.04 | 1.50 | 4.57 | 0.001 |  |
| IMS/APCAPS | Total cholesterol >200 mg/dL | Odds ratio | 2956 | GDQS | 1.09 | 0.98 | 1.20 | 0.065 | 0.170 |
| IMS/APCAPS | Total cholesterol >200 mg/dL | Odds ratio | 2956 | Operationalized GDQS | 1.06 | 0.96 | 1.17 | 0.408 |  |
| IMS/APCAPS | Triglycerides (mg/dL) | Estimated marginal mean | 2948 | GDQS | -0.10 | -2.36 | 2.16 | 0.821 | 0.280 |
| IMS/APCAPS | Triglycerides (mg/dL) | Estimated marginal mean | 2948 | Operationalized GDQS | 0.67 | -1.58 | 2.93 | 0.617 |  |
| IMS/APCAPS | Triglycerides ≥150 mg/dL | Odds ratio | 2948 | GDQS | 1.01 | 0.91 | 1.12 | 0.889 | 0.380 |
| IMS/APCAPS | Triglycerides ≥150 mg/dL | Odds ratio | 2948 | Operationalized GDQS | 1.04 | 0.94 | 1.15 | 0.636 |  |
| IMS/APCAPS | Waist circumference ≥80 cm | Odds ratio | 3035 | GDQS | 1.49 | 1.35 | 1.63 | <0.001 | 0.530 |
| IMS/APCAPS | Waist circumference ≥80 cm | Odds ratio | 3035 | Operationalized GDQS | 1.51 | 1.37 | 1.65 | <0.001 |  |
| IMS/APCAPS | BMI <18.5 kg/m^2^ | Odds ratio | 2961 | GDQS | 0.82 | 0.75 | 0.90 | <0.001 | 0.14 |
| IMS/APCAPS | BMI <18.5 kg/m^2^ | Odds ratio | 2961 | Operationalized GDQS | 0.78 | 0.72 | 0.85 | <0.001 |  |
| IMS/APCAPS | Hemoglobin (g/dL) | Estimated marginal mean | 3041 | GDQS | 0.01 | -0.05 | 0.08 | 0.610 | 0.340 |
| IMS/APCAPS | Hemoglobin (g/dL) | Estimated marginal mean | 3041 | Operationalized GDQS | -0.01 | -0.07 | 0.05 | 0.947 |  |
| IMS/APCAPS | Hemoglobin <12 g/dL | Odds ratio | 3041 | GDQS | 1.01 | 0.94 | 1.09 | 0.819 | 0.700 |
| IMS/APCAPS | Hemoglobin <12 g/dL | Odds ratio | 3041 | Operationalized GDQS | 1.02 | 0.95 | 1.09 | 0.631 |  |
| IMS/APCAPS | Mid-upper arm circumference <24.5 cm | Odds ratio | 3030 | GDQS | 0.78 | 0.72 | 0.84 | <0.001 | 0.280 |
| IMS/APCAPS | Mid-upper arm circumference <24.5 cm | Odds ratio | 3030 | Operationalized GDQS | 0.76 | 0.70 | 0.82 | <0.001 |  |
| IMS/APCAPS | Overall nutrient adequacy (EAR-based) (energy-adjusted) | Estimated marginal mean | 3041 | GDQS | 0.28 | 0.25 | 0.31 | <0.001 | 0.290 |
| IMS/APCAPS | Overall nutrient adequacy (EAR-based) (energy-adjusted) | Estimated marginal mean | 3041 | Operationalized GDQS | 0.25 | 0.21 | 0.28 | <0.001 |  |
| IMS/APCAPS | Overall nutrient inadequate (EAR-based) (energy-adjusted) | Odds ratio | 3041 | GDQS | 1.79 | 1.65 | 1.94 | <0.001 | 0.270 |
| IMS/APCAPS | Overall nutrient inadequate (EAR-based) (energy-adjusted) | Odds ratio | 3041 | Operationalized GDQS | 1.67 | 1.55 | 1.81 | <0.001 |  |
| MVP | Body mass index (kg/m^2^) | Estimated marginal mean | 451 | Operationalized GDQS | -0.05 | -0.41 | 0.32 | 0.730 | 0.472 |
| MVP | Body mass index (kg/m^2^) | Estimated marginal mean | 451 | GDQS | 0.08 | -0.28 | 0.45 | 0.678 |  |
| MVP | Mid-upper arm circumference (cm) | Estimated marginal mean | 517 | Operationalized GDQS | 0.50 | 0.15 | 0.86 | 0.003 | 0.005 |
| MVP | Mid-upper arm circumference (cm) | Estimated marginal mean | 517 | GDQS | 0.86 | 0.52 | 1.20 | <0.001 |  |
| MVP | BMI ≥25 kg/m^2^ | Odds ratio | 451 | Operationalized GDQS | 0.99 | 0.77 | 1.27 | 0.716 | 0.797 |
| MVP | BMI ≥25 kg/m^2^ | Odds ratio | 451 | GDQS | 1.06 | 0.82 | 1.36 | 0.741 |  |
| MVP | BMI <18.5 kg/m^2^ | Odds ratio | 451 | Operationalized GDQS | 1.11 | 0.85 | 1.44 | 0.772 | 0.169 |
| MVP | BMI <18.5 kg/m^2^ | Odds ratio | 451 | GDQS | 1.00 | 0.76 | 1.30 | 0.644 |  |
| MVP | Hemoglobin (g/dL) | Estimated marginal mean | 554 | Operationalized GDQS | 0.41 | 0.22 | 0.59 | <0.001 | 0.134 |
| MVP | Hemoglobin (g/dL) | Estimated marginal mean | 554 | GDQS | 0.52 | 0.33 | 0.71 | <0.001 |  |
| MVP | Hemoglobin <12 g/dL | Odds ratio | 554 | Operationalized GDQS | 0.87 | 0.74 | 1.03 | 0.083 | 0.169 |
| MVP | Hemoglobin <12 g/dL | Odds ratio | 554 | GDQS | 0.84 | 0.71 | 1.00 | 0.026 |  |
| MVP | Mid-upper arm circumference <24.5 cm | Odds ratio | 517 | Operationalized GDQS | 0.89 | 0.74 | 1.06 | 0.112 | 0.060 |
| MVP | Mid-upper arm circumference <24.5 cm | Odds ratio | 517 | GDQS | 0.75 | 0.62 | 0.90 | <0.001 |  |
| MVP | Overall nutrient adequacy (EAR-based) (energy-adjusted) | Estimated marginal mean | 1624 | Operationalized GDQS | 0.46 | 0.39 | 0.53 | <0.001 | 0.169 |
| MVP | Overall nutrient adequacy (EAR-based) (energy-adjusted) | Estimated marginal mean | 1624 | GDQS | 0.51 | 0.45 | 0.58 | <0.001 |  |
| MVP | Overall nutrient inadequate (EAR-based) (energy-adjusted) | Odds ratio | 1624 | Operationalized GDQS | 0.39 | 0.34 | 0.45 | <0.001 | 0.084 |
| MVP | Overall nutrient inadequate (EAR-based) (energy-adjusted) | Odds ratio | 1624 | GDQS | 0.35 | 0.30 | 0.40 | <0.001 |  |
